# Mechanisms of subliminal response priming

**DOI:** 10.2478/v10053-008-0032-1

**Published:** 2008-07-15

**Authors:** Andrea Kiesel, Wilfried Kunde, Joachim Hoffmann

**Affiliations:** 1 Department of Psychology, Julius-Maximilians University of Würzburg, Germany; 1 Department of Psychology, Martin-Luther-University Halle-Wittenberg, Germany

**Keywords:** subliminal priming, priming mechanism, action trigger account

## Abstract

Subliminal response priming has been considered to operate on several stages, e.g. perceptual, central or motor stages might be affected. While primes’ impact on target perception has been clearly demonstrated, semantic response priming recently has been thrown into doubt (e.g. Klinger, Burton, & Pitts, 2000). Finally, LRP studies have revealed that subliminal primes evoke motor processes. Yet, the premises for such prime-evoked motor activation are not settled. A transfer of priming to stimuli that have never been presented as targets appears particularly interesting because it suggests a level of processing that goes beyond a reactivation of previously acquired S-R links. Yet, such transfer has not always withstood empirical testing. To account for these contradictory results, we proposed a two-process model (Kunde, Kiesel, & Hoffmann, 2003): First, participants build up expectations regarding imperative stimuli for the required responses according to experience and/or instructions. Second, stimuli that match these “action triggers” directly activate the corresponding motor responses irrespective of their conscious identification. In line with these assumptions, recent studies revealed that non-target primes induce priming when they fit the current task intentions and when they are expected in the experimental setting.

## SUBLIMINAL RESPONSE PRIMING

The question whether or not stimuli that do not enter awareness influence behaviour has been explored for more than 100 years. For example, Sidis reported in 1898 that subjects were able to guess numbers printed on distant cards with above-chance accuracy, in spite of their self-reported inability to make out what was printed. However, for a long time “proofs” of unconscious manipulations of behaviour were criticized for methodological reasons and a lively debate about the standards for the investigation of unconscious manipulation evolved (cf. Holender, 1986; Reingold & Merikle, 1993).

Only in the mid-nineties, Neumann & Klotz (1994; see also Klotz & Neumann, 1999) reported subliminal response priming (or masked priming) experiments that convincingly demonstrated the impact of non-consciously presented stimuli on behaviour. In subliminal response priming experiments, participants are usually required to perform a forced choice reaction time task with two response alternatives according to a supraliminally presented target. Prior to the target another stimulus, the so-called prime, is presented subliminally. Reaction times are shorter if the prime elicits the same response as the target stimulus to which participants respond (congruent prime). In contrast, reaction times are increased if the prime is incongruent, that is if it elicits a different response than the target. [Under specific timing conditions and probably restricted to specific masking conditions, the priming effect is reversed, i.e. primes assigned to the same response as the target then delay responding (Eimer & Schlaghecken, 1998; Lleras & Enns, 2004; Verleger, Jaśkowski, Aydemir, van der Lubbe, & Groen, 2004)]. Thus, the prime has an impact on behaviour. To ensure that prime presentation is indeed subliminal, presentation time is very short, 10 to 50 ms, and the primes are masked. Furthermore, the visibility of the primes is tested separately. For this visibility test, prime, mask and target are presented exactly as in the experimental trials, but participants are requested to either identify or to discriminate the primes. If performance in the visibility test does not exceed chance level, the prime is considered to be presented subliminally, that is, unconsciously.

Thus, subliminal priming is characterized by the following dissociation: On the one hand, the prime causes a congruency effect, that is, participants respond faster to the target after congruent primes compared to incongruent ones. On the other hand, the prime is not seen in a visibility test; participants are not able to discriminate the primes or they do not identify the prime above chance.

In response priming the prime-target congruency results from overlap regarding the assigned motor responses and, of course, the stimulus features and processes used to assign the stimuli to these responses. In addition, other forms of subliminal priming have been investigated, like e.g. semantic priming (Kiefer, 2002; Kiefer & Spitzer, 2000) or priming of mental operations (Mattler, 2003). Here the prime-target relationship is not defined at the response level but regarding other prime-target aspects which are not necessarily used to specify the motor response. The scope of the current paper is restricted to subliminal response priming and does not elaborate on these other forms of priming.

Currently, the method of subliminal response priming is well-established and has become an often used method to investigate the influence of unconsciously seen stimuli (Dehaene, Naccache, Le Clec`H, Koechlin, Mueller, Dehaene-Lambertz, van de Moortele, & Le Bihan, 1998; Dell’Aqua & Grainger, 1999; Greenwald, Draine, & Abrams, 1996; Klotz & Neumann, 1999; Neumann & Klotz, 1994). Accordingly, research interest has shifted from establishing the phenomenon of unconscious priming to investigating the mechanisms underlying this phenomenon (e.g., Ansorge & Heumann, 2003, 2006; Ansorge, Heumann, & Scharlau, 2002; Ansorge & Neumann, 2005; Damian, 2001; Dehaene, et al., 1998; Dell’Acqua & Grainger, 1999; Kiesel, Kunde, & Hoffmann, 2007; Klinger, Burton, & Pitts, 2000; Kunde, Kiesel, & Hoffmann, 2003, 2005; Mattler, 2003; Snodgrass, Bernat, & Shevrin, 2004).

## MECHANISMS OF SUBLIMINAL RESPONSE PRIMING

Subliminally presented primes usually speed up responding to a subsequent target if they are assigned to the same rather than a different motor response. Now, the interesting question is: How do the primes work? Or which stages of the target processing are influenced by the prime? Basically, at least three different processing stages can be differentiated: Priming may influence target processing at perceptual, central, or response-related stages.

## Perceptual processes

There are several findings that convincingly demonstrate priming at perceptual stages: Firstly, masked primes reduce the latency of detecting the subsequent target when prime and target are presented at the same location. This perceptual latency priming presumably occurs because the prime initiates a shift of attention to its location and thereby facilitates perceptual target processing (e.g. Scharlau, 2002, 2004; Scharlau & Ansorge, 2003; Scharlau & Neumann, 2003).

Secondly, there is evidence that primes facilitate sensory processing of the target because additionally to congruency effects, identical prime-target pairs have been shown to facilitate responding. For example, Bodner and Dypvik (2005) instructed participants to categorize target numbers as being odd or even by pressing left or right response keys. Prior to the target number, a prime number was presented and masked to prevent conscious prime perception. Primes could be either identical (1-1, 2-2, etc.), congruent (1-3, 2-4, etc.), or incongruent (1-4, 2-3, etc.) with the target. Participants responded faster if primes were identical to, compared to congruent or incongruent with, the targets (for similar results see Bodner & Masson, 1997; 2003). Thus, primes speed up sensory processing of a subsequently presented identical target stimulus. Furthermore, when primes and targets occur either as a number word or as an Arabic digit, responding is faster when the target is a perceptually identical repetition (e.g. 1 -> 1) rather than when prime and target are the same on a conceptual level but differ in their peripheral notation (e.g. 1 -> one; Koechlin, Naccache, Block, & Dehaene, 1999). Obviously, perceptual prime-target similarity facilitates target processing.

## Central processes

Subliminal response priming also has been thought to be influenced by central processes (Greenwald et al., 1996; Marcel, 1980, 1983). Within this framework, stimuli are supposed to be processed mandatorily up to a semantic level independently of whether they are presented consciously or unconsciously (as suggested in the late selection account of Deutsch & Deutsch, 1963). A subliminally presented prime speeds up responding to the target because the prime automatically spreads activation in its semantic network. If the subsequently presented target stimulus belongs to the same semantic network, the target is processed faster because of the pre-activation in the net (for a detailed description of the spreading activation account see Neely, 1991).

Recently, the impact of spreading activation in subliminal response priming has been seriously questioned by the observation that primes affected the processing of targets selectively regarding task-relevant features. For instance, Klinger et al. (2000) used affective word stimuli as primes and targets. Primes evoked congruency effects when subjects categorized targets as being positive or negative. However, they had no impact on target processing when subjects were instructed to make a lexical decision (word vs. non-word). Thus, positive or negative primes are effective merely when targets are categorized according to their affective value, but not when a lexical decision is required. Within the same line of argumentation, there are quite a remarkable number of studies demonstrating that priming is restricted to current task requirements (e.g. Ansorge et al., 2002; Bodner & Dypvik, 2005; Kunde et al., 2003; Schlaghecken & Eimer, 2004). 

Thus, there are serious doubts as to whether semantic processing of subliminally presented stimuli and spreading activation in the semantic network is mandatory and therewith whether subliminal response priming is based on influences on central processing stages (for different opinions see Klauer, Musch, & Eder, 2005; Reynvoet, Gevers, & Caessens, 2005). On the contrary, prime processing seems to be determined by current task affordances and only information that is relevant for the required behavior has an impact in response priming studies.

## Response processes

Subliminal primes have the power to eventually trigger motor activation. The strongest evidence available comes from studies measuring LRP (lateralized readiness potentials). For example, Dehaene and colleagues reported that subliminal primes trigger LRPs, indicating a covert activation of the prime-related response (Dehaene, et al., 1998; for similar observations see Eimer & Schlaghecken, 1998; Leuthold & Kopp, 1998; Verleger et al., 2004). Pre-activation of the prime-related response facilitates responding to the subsequently presented target stimulus if the target requires the same response as the prime. In contrast, if the target requires the opposite response, responding is slowed down because the inappropriate prime-induced response activation hinders it. Thus, it is widely accepted that subliminally presented stimuli prime motor responses.

The question is, however, which processes are going on that enable the primes to trigger motor processes. Two extreme positions can be identified: The first one re-activates the idea of semantic prime processing and assumes that “unconscious primes activate motor codes through semantics” (Reynvoet, Gevers, & Caessens, 2005, p. 991). Thus, primes are submitted to the same semantic categorization procedures as conscious targets. And only after being categorized they activate the category-assigned motor response (see also Dehaene et al., 1998). However, in contrast to spreading activation accounts, primes influence target processing only if they belong to task-defined response categories. A mere semantic relatedness between prime and target does not suffice to influence target processing in a response priming paradigm.

Alternatively, it has been argued that the processing of unconscious stimuli is not elaborated. Instead, unconscious primes might activate responses by acquired S-R links between conscious target stimuli and motor responses. Later in the experiment, when a stimulus is shown as a subliminal prime that matches a stimulus one has already responded to, its associated response is retrieved (Abrams & Greenwald, 2000; Damian, 2001). This view denies the possibility of a deep analysis of unconscious stimuli and instead explains unconscious response priming by “simpler”, direct S-R links.

To decide between both accounts, one can consider whether stimuli that are never presented as targets (i.e. non-target primes) induce priming effects. It is not possible to acquire S-R mappings for primes that were never presented as targets. Thus, priming effects by non-target primes are crucial: If non-target primes remain ineffective despite their fit to the current task instructions, response priming is restricted to acquired S-R mappings. If, however, non-target primes cause response priming when they fit the current task context, subliminally presented primes are analyzed according to current task requirements.

Unfortunately, the existing evidence is contradictory. In some studies only target-primes, but not non-target primes, caused congruency effects (e.g. Abrams & Greenwald, 2000; Damian, 2001). In contrast, Naccache and Dehaene (2001) found that non-target primes caused congruency effects to a similar extent as target-primes (see also Greenwald, Abrams, Naccache, & Dehaene, 2003). 

To account for these contradictory results, we suggest a two-process model that will be described in detail in the following section. This model can be considered as an elaboration of the direct parameter specification account by Neumann (1990; see also Neumann & Klotz, 1994; Klotz & Neumann, 1999). The direct parameter specification account assumes that unconsciously registered information can specify an open parameter “if all parameters of the to-be-executed action have already been specified when the stimulus appears, except for those that will be specified by the stimulus itself” (Neumann & Klotz, 1994; p. 144). That is, if action planning has occurred, a subliminally presented prime stimulus can evoke motor responses by specifying the open parameter (e.g. performing a key press with left vs. right index finger) of a response. The aim of our model is to elaborate on how such a direct specification of parameters might work and under which circumstances it takes place.

## Action trigger account

**Figure 1.**
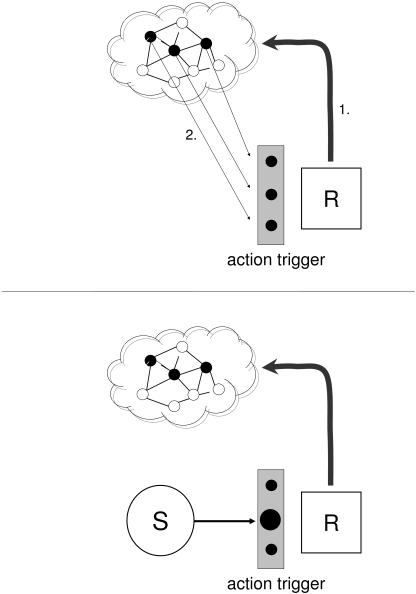
Schematic illustration of the two stages proposed by the action trigger account. Upper panel: Memory representations of those environmental events that shall prompt a specific motor response (1.) are recollected to specify action triggers (2.). Lower panel: Online processing is restricted to comparing whether a stimulus fits existing action triggers. Stimuli that correspond to the release conditions automatically trigger the related response.

Our account is based on the assumption that subliminally presented stimuli trigger responses neither because of semantic analysis nor because of acquired S-R links. Instead, primes trigger responses to the extent they fit existing action release conditions, which we termed action triggers. In a first processing step, such action triggers are specified according to expected or experienced task demands. In this step, participants recollect memory representations of those environmental events that subsequently shall prompt a specific motor response (see Figure 1). Thus, participants categorize to-be-expected imperative stimuli in appropriate and non-appropriate release conditions for the task-defined response alternative. Online stimulus processing, the second processing step, is then restricted to comparing whether the incoming stimulus fits an existing action trigger. If a stimulus matches to the release conditions of an action trigger, the related action is automatically activated (causing congruency effects if the stimulus was a prime).

To illustrate these ideas, consider the following example: Participants are instructed to categorize digits as being smaller or larger than five with the mapping left – smaller than 5 and right – larger than 5. The codes of the numerals 1, 2, 3 and 4 are then appropriate release conditions for pressing a left response key. Of course, representations of these numerals certainly encompass semantic information. For example, for the specific numeral 4 this might be information such as “four is smaller than 5 but larger than 3”, “a car has four wheels”, “four persons are needed to play a quartet”. But representations of trigger events are also associated with specific perceptual information, such as “what does the 4 look like when written as an Arabic digit or when printed on a dice?” We conjecture that semantic features can be used to select an event as an action trigger, that is, semantic features can be used to specify the release conditions for a response. But for subsequent response activation, a match with early available perceptual features is important. Only stimuli that perceptually resemble release conditions trigger responses; semantic similarity does not induce motor activation.

Underlying this approach is the idea of task preparation: The ultimate reason to prepare for a task is to bypass some of the slow and effortful semantic operations that would become necessary when the event was encountered unexpectedly. In other words, a subsequent stimulus is processed to an extent that suffices to detect a match with an appropriate action trigger, which is conceivably detected more quickly when based on perceptual rather than semantic features (for similar ideas see Ach, 1905; Bargh, 1989; Exner, 1879; Hommel, 2000; Neumann, 1990).

The action trigger approach allows for a considerable degree of flexibility regarding the origin of response priming effects. In fact, this approach more or less includes features of all the other accounts reviewed before. It includes perceptual facilitation. However, what counts is not the perceptual match of primes and subsequent targets but the perceptual match of primes with pre-specified trigger conditions. It also includes semantic processing. However, semantic processing is assumed to occur offline when environmental events are specified as adequate release conditions. Additionally, of course it assumes response activation which occurs if stimuli fit the release conditions of the action trigger. The entire idea of action triggers (and several comparable ideas in cognitive psychology) bears on the need to enable prompt responding in a more or less predictable environment. This approach might appear as eclectic or vague, but we believe that such an eclectic approach is indeed needed to explain the contradictory evidence on unconscious priming, that other accounts alone, fail to explain.

## Transfer of priming to unseen stimuli

As noted above, it seems particularly important to clarify when priming transfers to unseen stimuli and when it does not. According to the action trigger approach such a transfer of priming has its origins in the specification of action triggers. A transfer occurs if this stimulus event is considered as an action trigger. How can an event that is not experienced as a target become an action trigger? This might happen if it is closely related to the stimuli that are recollected as trigger events because they are experienced as stimuli. Thus, transfer of priming to unseen stimuli may occur due to semantic relatedness between primes and targets, but it is not the prime that semantically pre-activates the target. Instead, the seen targets lead to an inclusion of unseen stimulus events in the set of action triggers.

The action trigger approach may account for the observation of non-target priming by Naccache and Dehaene (2001) . Consider that participants had to judge whether digits are smaller or larger than 5, and in the experiments faced the numbers 1, 4, 6, and 9. In these conditions the numbers 1 and 4 become selected as action triggers for the left response and the numbers 6 and 9 become selected as action triggers for the right response. All we know about the mental representation of numbers tells us that they form an intimately integrated representation that is often described as a mental number line (Galton, 1880; Göbel, Walsh, & Rushworth, 2001). If now the numbers 1 and 4 have become selected as triggers for a left response, it seems likely that the mentally enclosed numbers 2 and 3 might enter the same trigger set when there is no obvious harm in doing so. If this “incidental recollection” account is correct, it should be less likely that unseen stimuli become considered as action triggers when the semantic distance to the experienced trigger events is larger. This seems to be so. When the digits 3 and 4 are used as targets (and selected as action triggers) the neighboring, but not enclosed, numbers 1 and 2 exert essentially no priming effect (Kunde et al., 2003, Exp. 2). 

Conceivably, a recollection as action trigger is less likely, the less closely the mental presentations of these events are related to experienced trigger events. Therefore, it is presumably much harder to obtain transfer of priming for stimuli that share a more arbitrary criterion (like pleasantness in the study of Abrams & Greenwald, 2000, or size in the study of Damian, 2001) than for digit stimuli that are tightly related. To explore this issue we conducted a study where the participants judged whether the object denoted by a target word was smaller or larger than a football. Obviously, the number of potential objects smaller or larger than a football is quite large and they might be associated to each other on an almost infinite number of semantic dimensions (such as evaluative content, being animate, size, etc.). Thus, an activation of a concept that is somehow linked to a target word seems much less likely than in the case of the tight-knit numbers. In fact, with an analogue version of the number experiment reported above with only four target words we found no spread of priming to unseen stimuli. Yet, when the number of consciously encountered targets increased to forty words, there was a transfer of priming to novel prime words (Kiesel, Kunde, Pohl, & Hoffmann, 2006). We conjecture that with such a large set of target words the participants consider other words as potential targets as well. For example, after having experienced the words “knife”, “mug” and “cup” it seems possible that the word “spoon” becomes recollected as well, either because participants are intentionally expecting this word, or because the preceding target words activated codes of this word as well. By contrast, there is no reason to intentionally prepare for other words after it has become clear that only four words are used as targets, and also a collateral activation from these target words to other unseen words is unlikely. Thus, this finding is in line with the studies of Damian (2001) and Abrams and Greenwald (2000) . In those cases no congruency effects for non-target primes were observed while small target sets, consisting of 12 and 16 exemplars respectively, were used. 

The action trigger approach can account for hitherto contradictory evidence on unconscious priming. Additionally, it enables a further prediction: Transfer to unseen stimuli should be restricted to exemplars of stimuli that are related to the targets. Conversely, no further transfer, for example to non-experienced notations, would be predicted. Action triggers are assumed to be set up in a way that allows an easy check on whether stimuli match the release conditions based on early available features. There is no reason to build up action triggers for notations that were never experienced. Given that primes are not processed semantically, but just according to whether they match the release condition, priming should be restricted to experienced target notations. Such a result was observed when participants categorized digit stimuli as being smaller or larger than 5 (Kunde, et al., 2003, Exp. 4). Targets were the numbers 1, 4, 6 and 9, which were presented in Arabic notation for one group of participants and as number words for another group of participants. Priming transferred to unseen stimuli in the experienced notation, but there was no transfer to those in the alternative notation.

## IMPLICATIONS FOR THE STUDY OF CONSCIOUSNESS

When investigating how unconscious stimuli are processed, there is an admittedly very ambitious goal: By elaborating the possibilities and boundaries of unconscious stimulus processing, we hope to draw conclusions about the functionality of consciousness. Cases in which subliminally presented stimuli remain ineffective are of special interest because they demonstrate the functionality of consciousness.

Evidence discussed in the current paper shows that regarding the processing stages for stimuli, consciousness is clearly not necessary to speed up perceptual processes and it is also not necessary to activate motor processes. In contrast, within experimental conditions when investigating response priming access to more abstract memory codes, semantic processing seems to be restricted to consciously presented stimuli (for similar conclusions see Holender & Duscherer, 2004).

For future research it is a challenge to identify more processes that are restricted to conscious stimuli. For example, higher order processes like executive functions might be bound to consciousness. Initial evidence in this direction was brought forward by Kunde (2003) . He explored whether participants adapt to conflict that was induced by subliminally and supraliminally presented prime stimuli. Conflict adaptation was restricted to cases where conflict was evoked by supraliminal stimuli. Subliminally presented stimuli evoked conflict by means of response congruency effect, but participants did not adapt to this subliminally evoked conflict. At first glance, the results of Jaśkowski, Skalska and Verleger (2003) might contradict this conclusion. They observed that the effect size of subliminal response priming depended on the ratio of incongruent and congruent prime stimuli. Priming effects were weaker if incongruent primes were presented more frequently than congruent primes. However, to account for this finding, they do not assume a conflict adaptation mechanism evoked by subliminally presented stimuli. Instead, they propose that the openly observable error frequency (which is higher if incongruent primes are presented more often) causes participants to act more cautiously and to prevent unconscious prime processing. Thus, their reasoning is completely in line with the assumption that offline control determines whether and to what degree subliminally presented primes become effective. Also, it does not contradict the assumption that consciousness is a prerequisite for executive control processes (see also Mayr, 2004; McCormick, 1997). However, future research is needed to clarify the necessity of consciousness on executive functions.
